# Stink bug species composition and risk of economic damage in the southeastern soybean cropping systems

**DOI:** 10.1093/ee/nvaf124

**Published:** 2025-12-04

**Authors:** Sujan Panta, George G Kennedy, Dominic D Reisig, Rachel A Vann, Benjamin L Aigner, Kyle Matthew Bekelja, Sean Malone, Hélène B Doughty, Tim B Bryant, Thomas P Kuhar, Anders S Huseth

**Affiliations:** Department of Entomology and Plant Pathology, North Carolina State University, Raleigh, NC, USA; Plant Sciences Initiative, North Carolina State University, Raleigh, NC, USA; Department of Entomology and Plant Pathology, North Carolina State University, Raleigh, NC, USA; Department of Entomology and Plant Pathology, North Carolina State University, The Vernon James Center, Plymouth, NC, USA; Plant Sciences Initiative, North Carolina State University, Raleigh, NC, USA; Department of Crop and Soil Sciences, North Carolina State University, Raleigh, NC, USA; Department of Entomology, Virginia Polytechnic Institute and State University, Blacksburg, VA, USA; Department of Entomology, Alson H. Smith Jr. Agricultural Research and Extension Center, Virginia Polytechnic Institute and State University, Winchester, VA, USA; Tidewater Agricultural Research and Extension Center, Virginia Polytechnic Institute and State University, VA, USA; Eastern Shore Agricultural Research and Extension Center, Virginia Polytechnic Institute and State University, Painter, VA, USA; Tidewater Agricultural Research and Extension Center, Virginia Polytechnic Institute and State University, VA, USA; Department of Entomology, Virginia Polytechnic Institute and State University, Blacksburg, VA, USA; Department of Entomology and Plant Pathology, North Carolina State University, Raleigh, NC, USA; Plant Sciences Initiative, North Carolina State University, Raleigh, NC, USA; Department of Entomology, Michigan State University, East Lansing, MI, USA

**Keywords:** stink bugs, soybean, economic threshold, ecoregion, latitudinal gradient

## Abstract

Stink bugs (Hemiptera: Pentatomidae) have emerged as an important pest species complex in soybean production systems across the southeastern United States. Changing cropping practices and climatic conditions are reshaping the stink bug communities in the region. Understanding community differences will be important to tailor integrated pest management programs sensitive to variation in species composition. In this 3-year study (2022-2024), we characterized stink bug diversity and abundance in 154 commercial soybean fields distributed across 3 soybean-producing ecoregions (Coastal Plain, Piedmont, and Mountains) in 2 southeastern states, North Carolina and Virginia. Standardized 25-sweep samples were collected at 10 locations per field during the soybean reproductive stages. Field-level samples were used to evaluate the probability of exceeding the recommended economic threshold for damage. We observed differences in stink bug community composition and spatial variation in the distribution of common stink bug species across the ecoregions. Additionally, the risk of soybean fields exceeding the recommended economic threshold differed across the ecoregions, with the Mountain region at the greatest risk. This result highlights the importance of regionally specific scouting and management recommendations that are sensitive to species composition differences. This work also provides a benchmark to assess range shifts of stink bug species in North Carolina and Virginia.

## Introduction

The stink bug complex is one of the most important pest groups of soybean—*Glycine max* (L.) Merrill—in the United States (McPherson and McPherson 2000, Kamminga et al. 2012, Owens et al. 2013, Musser et al. 2024). Stink bugs are particularly problematic in the southeastern United States, where they causes injury to both field and specialty crops. Expert opinion during 2023 ranked stink bugs second and first most damaging insect pests of soybean in North Carolina and Virginia, respectively (Musser et al. 2024). In this year, combined stink bug-related soybean yield losses and management costs in these states were estimated at 18% and 32%, respectively (Musser et al. 2024). Stink bugs cause yield loss through direct feeding on pods and seeds, reduced seed quality, and increased susceptibility to secondary pathogens (Daugherty et al. 1964, Russin et al. 1988, McPherson and McPherson 2000, Medrano et al. 2009). Additionally, severe stink bug infestations have been linked to delayed maturity, commonly referred to as “green stem syndrome,” which is a physiological condition that complicates harvest efficiency (Boethel et al. 2000).

Historically, stink bugs in the southeastern region were of secondary importance relative to more severe lepidopteran pests (eg *Helicoverpa zea* Boddie, *Chloridea virescens* F., *Chrysodeixis includens* Walker) (Huseth et al. 2021). A statewide soybean arthropod survey in North Carolina in 1976 reported a minimal need to control the 2 observed endemic species, green stink bug (*Chinavia hilaris* Say) and brown stink bug (*Euschistus servus* Say) (Deitz et al. 1976). Several other species not observed in that survey are now common in southeastern soybean production systems, including invasive brown marmorated stink bug (*Halyomorpha halys* Stål) (Leskey et al. 2012, Bakken et al. 2015, Aigner et al. 2017, Ogburn et al. 2022) and southern green stink bug (*Nezara viridula* L.) with a range that now extends into southeastern North Carolina. These observations suggest a shift in stink bug community composition has occurred since the 1970s.

Several factors may contribute to stink bug community change, including shifting soybean agronomic practices (Vann et al. 2021), adoption of cotton and corn expressing *Bt* reduced the use of broad-spectrum insecticides that previously controlled secondary pests such as stink bugs (Fernandez-Cornejo et al. 2014), and variable climatic conditions (Yukawa et al. 2009, Ingram et al. 2013, Chen et al. 2023). Although current management recommendations do not differentiate among species, clear insecticide susceptibility differences may require species-specific guidelines for soybean in the future (Panta et al. 2025). Understanding stink bug community composition in current soybean production systems will be important for effective and regionally relevant management information.

Recent studies have documented similar changes in stink bug ranges and infestation levels in other United States soybean growing regions (Tillman 2010, Herbert and Toews 2011, Temple et al. 2013b, Koch and Pahs 2014, Pezzini et al. 2019). For example, *H. halys*, introduced from Asia in the mid-1990s (Hoebeke and Carter 2003), is now an established economic pest across much of the United States, including North Carolina and Virginia (Leskey et al. 2012). In recent decades, the redbanded stink bug (*Piezodorus guildinii* Westwood) has become a yield-limiting species in mid-southern states, eg, in Arkansas and Louisiana (Temple et al. 2013b). Paul et al. (2024) used species distribution models to explore potential range expansion of *P. guildinii* and found that habitats in North Carolina and Virginia could be suitable for this pest in the future. More broadly, *Euschistus heros* Fabr. has emerged as a major stink bug pest of Brazilian soybeans over fewer than 5 decades (Panizzi 2015). These trends suggest a change in stink bug communities in response to changing climatic and agronomic conditions (Panizzi and Lucini 2016). Understanding the rate of change over time may improve regionally specific soybean pest management practices in the future.

There is now considerable anecdotal evidence that region-specific stink bug communities have changed since the last comprehensive soybean arthropod survey nearly 50 years ago (Deitz et al. 1976). North Carolina and Virginia soybean production occurs in 3 ecologically distinct regions: Coastal Plain, Piedmont, and Mountains (ecoregions hereafter). Extending from near sea level to moderate elevations (∼600 meters above sea level [MASL]), soybean fields in these ecoregions differ in climatic factors that can affect the distribution of insect populations such as rainfall, maximum and minimum temperatures, and extreme weather (eg drought) (Robinson 2005, Ingram et al. 2013). Native vegetation and cropping systems surrounding soybean fields also vary markedly among the 3 ecoregions. Specifically, the Coastal Plain is a relatively simplified landscape mosaic with large and contiguous fields dominated by crops, eg, wheat (*Triticum aestivum* L.), corn (*Zea mays* L.), cotton (*Gossypium hirsutum* L.), and soybean. In contrast, the Mountain ecoregion has smaller fields that are embedded within a matrix of non-crop habitat. It is unclear how variable the stink bug complex is among these distinct ecoregions, but there are likely differences. For example, *H. halys* is prevalent in North Carolina Mountain and Piedmont soybeans, but not in Coastal Plain soybeans (Bakken et al. 2015).

The goal of this study was to document the stink bug complex in each ecoregion, provide current data for management, and baseline to estimate regionally specific rates of change in the future. To do this, we conducted a multi-year, statewide survey spanning the 3 ecoregions in North Carolina and Virginia to document the current stink bug species composition, their geographic distribution, and abundance. We hypothesized that stink bug community composition associated with soybean would vary across the ecoregions in response to typical environmental conditions, such that tropical species like *N*. *viridula* would be more prevalent in the warmer Coastal Plain fields, whereas temperate species (ie, *H. halys*) would be more abundant in the cooler Mountain region. We used standardized sampling protocols consistent with soybean scouting recommendations to determine the probability of exceeding economic threshold across ecoregions. Understanding the current composition of stink bug communities provides a basis for designing regionally tailored pest management recommendations and prevention of undue economic loss.

## Materials and Methods

### Survey Extent and Sampling

**Fig. 1.**
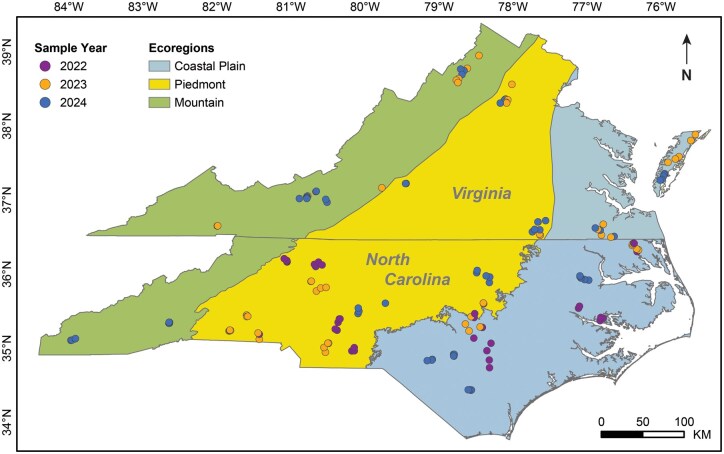
Soybean field locations sampled for stink bug survey in North Carolina and Virginia over 3 years in 3 ecoregions.

Stink bug surveys were conducted over 3 years in North Carolina (2022-2024) and 2 in Virginia (2023-2024) (Fig. 1). The soybean fields selected for this survey were distributed across the 3 ecoregions shared by both states—the Coastal Plain, Piedmont, and Mountains—to capture variation in stink bug community composition associated with differences in elevation, climate and land use. The sampled soybean fields spanned a geographic range of 500 km in latitude (34.51125°N to 39.03191°N), 945 km in longitude (-75.45079°W to -83.88667°W), and elevation from 5.7 to 662 MASL.

Across states and years, a total of 154 distinct soybean fields were sampled. In North Carolina, 34 in 2022, 30 in 2023, and 34 in 2024 (*n* = 98 different fields). In Virginia, sampling was conducted in 2023 and 2024, with 28 fields surveyed in each year (*n* = 56 different fields). All surveyed fields were located on commercial farms that represented typical soybean production system in the region. Soybean varieties, planting dates, and management practices were representative of respective regions. The sampling targeted soybeans during reproductive growth stages, when stink bugs are more abundant and can cause the greatest damage to soybeans (McPherson and McPherson 2000). Timing of these growth stages, which varied in time between years and ecoregions, is strongly influenced by different planting dates and crop rotations (Vann et al. 2021). In North Carolina, sampling was conducted from August to October in 2022, September in 2023, and from July to October in 2024. In Virginia, soybean fields were sampled between August and September in both years.

A standardized sweep net protocol modified from Pezzini et al. (2019) was used. Sampling was conducted during the reproductive growth stages from R3 (beginning pod) to R8 (maturity) to capture peak stink bug activity, with growth stages determined following Fehr et al. (1971). Each field was visited once, and the specific growth stage was recorded at sampling. Samples were evenly distributed around the perimeter of each soybean field within 5-10 m of the field edge to account for the edge-based tendency of stink bugs (Reay-Jones 2010). Samples comprised 10 sample units of 25 sweeps per unit in all except 3 fields (1 with 8 and 2 with 9 sample units). Sampling was avoided during precipitation events and in fields with known insecticide applications. Each sample location was georeferenced at the time of collection. Because field sizes differed, distance between sample units varied. After each set of 25 sweeps, sweep net contents were transferred into pre-labeled plastic bags (26 cm × 28 cm, Hefty Fast-Pak, ULINE, Atlanta, GA, USA) and stored in a cooler. These sample bags were stored at -20°C until sorting and identification.

Each sample unit was processed in the laboratory, where adult and immature stink bugs were separated for identification. Adult stink bugs were identified at the species or subspecies level, while nymphs were identified at either the genus or species level. Specifically, because of their morphological similarity, nymphs of the genera *Euschistus* and *Thyanta* were identified only to genus (eg Pezzini et al. 2019). Adult and immature stink bug identification was conducted following established taxonomic keys and diagnostic references (Decoursey and Esselbaugh 1962, Cuda and Mcpherson 1976, Rider and Chapin 1992, McPherson and McPherson 2000, Esquivel et al. 2009, Paiero et al. 2013, Packauskas 2012).

### Statistical Analysis

North Carolina and Virginia have contiguous soybean production systems and use similar stink bug management tactics (McPherson and McPherson 2000, Kamminga et al. 2012). Thus, data from both states were pooled by year and ecoregion across states for analysis. A total of 59, 49, and 46 soybean fields were sampled from the Coastal Plain, Piedmont, and Mountain ecoregions, respectively. Counts of adults and nymphs were summed by species for each field prior to analysis. To avoid overinflating estimates of diversity, richness, and geographic distributions, subspecies were combined to their respective parent species, as they share similar biology and ecology.

#### Species Composition, Diversity, and Richness

We analyzed the stink bug community compositions across the ecoregions using transformation-based principal component analysis (tb-PCA). Species abundance data were Hellinger transformed to reduce the influence of highly abundant species and the effect of double-zeros (Legendre and Gallagher 2001). Furthermore, we used permutational multivariate analysis of variance (PERMANOVA) based on Bray-Curtis dissimilarities to evaluate differences in community composition among ecoregions (Anderson 2017). Test of dispersion confirmed the assumption of homogeneity of multivariate dispersion (*F *= 0.36; df = 2, 153; *P *= 0.69). Both tb-PCA and PERMANOVA were conducted using the package *vegan* (Oksanen et al. 2025) in R (R Core Team 2025). A PCA biplot was used to visualize variation in community structure among ecoregions and to examine the contribution of individual species using the *ggplot2* package in R (Wickham 2011).

We estimated species richness using the Chao1 species richness estimator, a robust non-parametric method (Chao and Lin 2012, Chao and Chiu 2016). The Chao1 estimator provides a lower-bound estimate of true richness by accounting for both observed species, as well as singletons and doubletons (Goetelli and Colwell 2010, Chao and Lin 2012, Chao and Chiu 2016). The Shannon diversity index was estimated for each field location. The Shannon diversity index is a robust diversity metric that measures the uncertainty of predicting the species identity of a randomly chosen individual and places more emphasis on species richness (Shannon 1948, DeJong 1975). Both the Chao1 species richness and the Shannon diversity index were estimated using the *vegan* package in R.

**Fig. 2.**
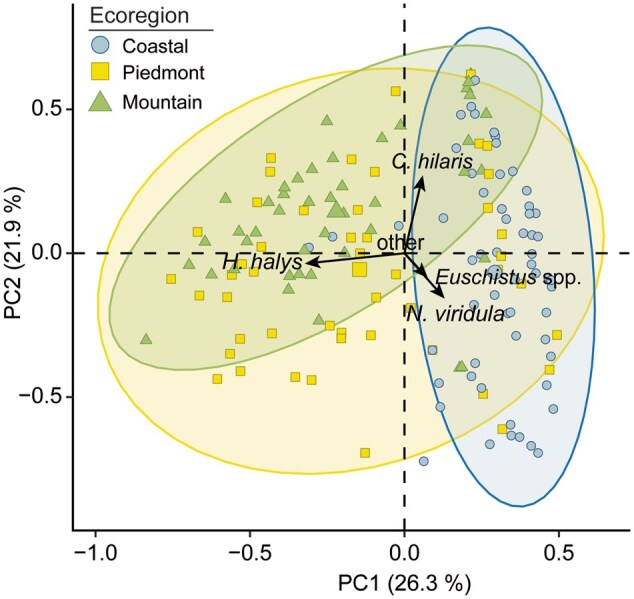
Transformation-based principal component biplot of stink bug community composition in soybean production system across the ecoregions. The length and orientation of species vectors (arrow) indicated both their influence on overall community assemblage variation and association with ecoregions. The colored ellipses represent the 95% confidence intervals for each ecoregion, illustrating the typical spread and overlap of each region. Only top 4 contributing species were displayed on the plot and the remaining species were pooled into the other category.

Four species dominated samples in the study: *C*. *hilaris*, *N*. *viridula*, *H*. *halys*, and *Euschistus* complex. These 4 taxa were also key contributors to the regional difference in species assemblage in the tb-PCA analysis (Fig. 2). As a result, we evaluated the spatial variability of these 4 taxa to understand their distribution across the study extent. Adults and nymphs were combined for each species, as both share a similar feeding guild, can cause significant soybean damage, and are typically combined for spray threshold decisions (McPherson and McPherson 2000, Reisig 2021). All *Euschistus* species share similar ecological niches and were grouped and analyzed as a single taxonomic unit (*Euschistus* complex) (Aldrich et al. 1991, McPherson and McPherson 2000, Schaefer and Panizzi 2000, Pezzini et al. 2019). To account for abundance variability, we standardized abundance for each species by calculating the proportion of each stink bug species relative to the total stink bug community at each field (relative abundance hereafter) and modeled this metric as a response variable. This allowed us to assess species-specific distribution patterns independent of total stink bug density.

#### Modeling Approach

Generalized linear mixed models were used to analyze the stink bug diversity, richness, and their relative abundance as response variables. Ecoregions and geographic parameters (latitude, longitude, and elevation) were modeled as predictors. Latitude and longitude were recorded in decimal degree format using the ArcGIS Field Maps applications (Version 25.1.0, Esri, Redlands, CA, USA) during the sampling visit. Elevation data were extracted from the North America Digital Elevation Model (DEM) published by the United States Geological Survey (https://www.usgs.gov/faqs/what-a-digital-elevation-model-dem), using ArcGIS Pro (Version 2.9, Esri, Redlands, CA). The DEM provides elevation information in raster form with a spatial resolution of 30-arc seconds (∼760 m resolution at 35°N). In our study, elevation and longitude were positively correlated along an east to west gradient (*r *= 0.81; df = 152; *t* = -16.87; *P *< 0.01). Predictors with a variance inflation factor (VIF) greater than 5 suggest a potential multicollinearity (Chatterjee and Simonoff 2013, Marcoulides and Raykov 2019). We examined the VIF of all predictors and removed the predictor with the highest value, longitude (VIF = 14.75) in this case. This was reasonable because ecoregions capture the spatial habitat variation aligned along a similar east-west gradient, and elevation represents additional ecological relevance, such as temperature and precipitation variation (Sundqvist et al. 2013 and references therein). The final model for diversity, species richness, and relative abundance (response variables) included ecoregions, latitude, and elevation as fixed effects (predictors). Latitude and elevation were mean-centered to simplify the model fit using the scale function in base R. Sampling year was included as a random effect, and residual variance was grouped by year to account for temporal variation.

Shannon’s diversity index was modeled with a Gaussian distribution with an identity link function. Chao1 species richness was modeled with a log-normal error distribution with an identity link function. Relative abundance of *C*. *hilaris* and *H*. *halys* was analyzed using a beta distribution with a logit link function, while a log-normal distribution with an identity link function was used for the *Euschistus* complex. For *N*. *viridula*, more than 50% of fields did not record any individuals, resulting in a mix of zero and positive values. To account for the excess zeros, we used a generalized linear model using PROC GENMOD with the Tweedie distribution function (Tweedie 1984). The fit of each model was checked using model diagnostics plots. Significance among main effect levels was determined using Tukey-Kramer pairwise comparisons (*P *< 0.05). These analyses were performed in SAS (Version 9.4, Cary, USA).

#### Economic Threshold Risk

We evaluated the probability of soybean fields exceeding the economic threshold (ET) for stink bugs across our study extent. Only phytophagous stink bug species were included in the analysis. Total stink bug abundance (adult + nymphs) was calculated at the field level. Multiple phytophagous species and both plant feeding life stages were combined because threshold calculations use the average stink bug sample (typically collected by sweeping) pooled across all species in the region (Reisig and Huseth 2025). Furthermore, both life stages can cause significant damage and are controlled using a similar management tactic (McPherson and McPherson 2000).

A recommended economic threshold of 5 stink bugs per 15 sweeps for grain soybean (Reisig 2021) was used to classify each field as either above ET or below (binary response variable). To assess the risk of exceeding the ET, we fit a generalized linear mixed model with a binomial error distribution and a logit link function using the *glmmTMB* package in R (Brooks et al. 2017). Fixed effects were ecoregion, latitude, elevation, and sampling year to account for the spatial and temporal variation. All continuous predictors (latitude, elevation, and year) were mean-centered to improve model interpretability. We also included an interaction term between ecoregion and latitude to assess how the risk of exceeding ET varied across ecoregions by latitude. Model diagnostics were performed to examine the model fit using the *DHARMa* package (Hartig et al. 2025). Spatial autocorrelation was assessed using Moran’s I based on k-nearest neighbors and was not significant (Moran’s I = 0.001, *P *= 0.32). Predicted probabilities of fixed effects were obtained using the *emmeans* package in R (Lenth and Lenth 2018), and fitted estimates were generated across the latitude range for each ecoregion to assess the interaction effects. Significance of main effects was determined at *P *< 0.05, and Tukey-Kramer pairwise comparisons were used to test differences among the groups (*P *< 0.05).

#### Spatial Distribution of Soybean

To illustrate the pattern of soybean production intensity among the ecoregions, total soybean production area for 2024 was extracted from the publicly available United States Department of Agriculture, National Agricultural Statistics Service (USDA-NASS) Cropland Data Layer (CDL) (https://croplandcros.scinet.usda.gov). The CDL is a georeferenced, crop and non-crop land use raster data layer produced using satellite imagery and extensive agricultural ground reference data at 30 m × 30 m spatial resolution. The CDL layer was reclassified to soybean and non-soybean areas in ArcGIS Pro. A majority filter was used to eliminate mixed pixels and contiguous soybeans were converted to polygons using the *Convert Raster to Polygon* tool in ArcGIS Pro. Field centroids were calculated and merged with the ecoregion polygon layer to align with analyses above. Soybean area by ecoregion was calculated for each one-tenth degree of latitude (11 km) using the *Tabulate Area* tool in ArcGIS Pro and was summarized by ecoregion in R (R Core Team 2025).

## Results

Over a 3-year study period, a total of 17,140 stink bug individuals, including 4,784 adults, were collected. In total, 30 stink bug species and subspecies were identified (Table 1). The majority were phytophagous (*n* = 27), while 3 were predatory (Table 1). Five phytophagous species—including subspecies, *C. hilaris*, *H. halys*, *N*. *viridula*, *E. servus*, and *Euschistus tristigmus* (Say)—accounted for 90.20% of total adult stink bugs collected (Table 1). Across ecoregions, *C*. *hilaris* was the most abundant species, followed by the invasive *H*. *halys* (Table 1). Additionally, our study recorded *N*. *viridula* to 37°N latitude (southern Virginia), a result that was surprising given previous reports that their range only extended northward into southern NC counties. We also recorded large numbers of economically important *Euschistus* spp., including *E*. *servus* (Table 1). Subspecies recorded included *E. servus* (Say) and *E. servus euschistoides* (Vollenhoven), *E. tristigmus luridis* (Dallas), and *E. tristigmus* (Say) as well as *Thyanta custator* (Fabr.) and *T. custator accerra* (McAtee) (Table 1). Among predatory stink bugs, *Podisus maculiventris* (Say) accounted for an estimated 87% of the total adult predatory species (Table 1). We collected a total of 12,356 nymphs, with *C*. *hilaris*, *N*. *viridula*, *H*. *halys*, and *Euschistus* spp. accounting for 98.86% of the total (Table 2).

**Table 1.** Summary of adult stink bug abundance across the ecoregions from soybean production systems in North Carolina and Virginia over 3 years (2022-2024)

Stink bug species	Ecoregions				Relative abundance (%)
	Coastal Plain	Piedmont	Mountains	Total	
**Total adults**	1988	1260	1536	4784	100
**Subfamily: Pentatominae**					
***Chinavia hilaris* (Say)**	626	268	528	1422	29.72
***Halyomorpha halys* (Stål)**	42	582	718	1342	28.05
***Nezara viridula* (L.)**	838	106	16	960	20.07
***Euschistus servus servus.* (Say)**	222	102	52	376	7.86
***Euschistus tristigmus tristigmus.* (Say)**	61	34	48	143	2.99
***Thyanta custator accerra* McAtee**	34	23	46	103	2.15
***Euschistus servus euschistoides* (Vollenhoven)**	5	28	24	57	1.19
***Euschistus conspersus* Uhler**	7	20	25	52	1.09
***Euschistus ictericus* (L.)**	28	11	13	52	1.09
***Euschistus obscurus* (Palisot)**	17	13	2	32	0.67
***Oebalus pugnax* (F.)**	6	13	8	27	0.56
***Euschistus crassus* (Dallas)**	11	6	7	24	0.5
***Brochymena quadripustulata* (F.)**	2	4	17	23	0.48
***Thyanta calceata* (Say)**	3	7	8	18	0.38
***Euschistus tristigmus luridus* Dallas**	5	3	7	15	0.31
***Euschistus quadrator* Rolston**	3	8	4	15	0.31
***Euschistus variolarius* (Palisot)**	1	2	1	4	0.08
***Cosmopepla lintneriana* (Kirkaldy)**	1	2	0	3	0.06
***Thyanta custator* (Fab.)**	1	1	1	3	0.06
***Holcostethus limbolarius* (Stål)**	0	1	1	2	0.04
***Hymenarcys nervosa* (Say)**	1	1	1	3	0.06
***Murgantia histrionica* (Hahn)**	1	1	0	2	0.04
***Mormidea lugens* (Fabr.)**	2	0	0	2	0.04
***Coenus delius* (Say)**	0	1	0	1	0.02
***Euschistus politus* Uhler**	0	1	0	1	0.02
***Holcostethus fulvipes* Ruckes**	1	0	0	1	0.02
***Trichopepla articornis* Stål**	1	0	0	1	0.02
***Subfamily: Asopinae***					
***Podisus maculiventris* (Say)**	60	19	8	87	1.82
***Euthyrhynchus floridanus* (L.)**	7	3	0	10	0.21
***Stiretrus anchorago* (Fabr.)**	2		1	3	0.06

**Table 2.** Summary of stink bug nymph abundance across the ecoregions in North Carolina and Virginia over 3 years (2022-2023)

Stink bug	Stink bug nymph abundance				Relative abundance (%)
	Coastal Plain	Piedmont	Mountains	Total	
**Total nymphs**	5267	3787	3302	12356	100.00
***Chinavia hilaris***	1366	1406	1532	4304	34.83
***Nezara viridula***	2655	384	119	3158	25.56
***Halyomorpha halys***	503	1288	1191	2982	24.13
***Euschistus* spp.**	688	673	410	1771	14.33
***Podisus maculiventris***	37	26	21	84	0.68
***Thyanta custrator***	13	6	16	35	0.28
***Oebalus pugnax***	5	4	13	22	0.18

Nymphs of *Euschistus* spp. were identified to genus level and presented in this table.

### Species Diversity and Abundance

Analysis using transformed-based PCA demonstrated that the first 2 principal components explained 48.2% of the total variation observed (Fig. 2). The result of PERMANOVA showed ecoregion has a significant effect on stink bug community compositions (*F *= 6.50; df = 2, 151; *P *= 0.001; *R*^2^ = 0.08), with variation most tightly clustered in the Coastal Plain region indicating species composition were consistent across sites. The Piedmont region was more dispersed, meaning that sites varied more, reflecting a transitional zone between the Coastal Plain and the Mountain. In PCA, most stink bug species clustered around the ordination centroid, indicating broad distribution across the ecoregions (Fig. 2). In contrast, *C*. *hilaris*, *N*. *viridula*, *Euschistus* complex, and *H*. *halys* were positioned away from the centroid, suggesting that these species contributed to region-specific differences in species assemblage (Fig. 2).

Shannon’s diversity did not differ among ecoregions (*F *= 2.02; df = 2, 149; *P *= 0.14), suggesting they were similarly diverse (Table 3). Similarly, elevation did not influence stink bug diversity (*F* = 1.99; df = 1, 149; *P *= 0.16). In contrast, latitude had a significant effect on stink bug diversity (*F* = 7.71; df = 1, 149; *P *< 0.01). There was a negative association with latitude, indicating stink bug diversity declines along a south-to-north gradient (Table 3).

**Table 3.** Parameter estimates (±SE) for predictors for stink bug diversity, species richness, and relative abundance of 4 common observed stink bug species

Predictor	Biodiversity		Relative abundance			
	Diversity	Richness	*C. hilaris*	*H. halys*	*Euschistus* spp.	*N. viridula*
**Ecoregions**						
**Coastal Plain**	0.891 (0.085)	1.406 (0.125)	-0.523 (0.177)	**-1.488 (0.256)b**	-1.334 (0.166)	-2.945 (0.477)
** Piedmont**	1.086 (0.063)	1.617 (0.092)	-0.891 (0.136)	**-0.356 (0.139)a**	-1.625 (0.123)	-3.239 (0.293)
** Mountain**	1.123 (0.122)	1.575 (0.177)	-0.647 (0.234)	**-0.903 (0.209)ab**	-1.759 (0.215)	-3.677 (0.684)
**Latitude**	-**0.092 (0.033)**	**-0.173 (0.049)**	-0.089 (0.076)	-0.100 (0.075)	**0.293 (0.062)**	**-0.676 (0.139)**
**Elevation**	-0.001 (0.0004)	-0.0001 (0.001)	0.230 (0.142)	0.001 (0.001)	-0.0005(0.001)	**-0.008 (0.003)**

The values reported in the table above are model estimates. The significance of continuous variables for latitude and elevation (*P *< 0.05) are highlighted in bold.

Chao1 species richness estimate did not differ among ecoregions (*F* = 1.18; df = 2, 149; *P *= 0.31), or with elevation (*F *= 0.05; df = 1, 149; *P *= 0.83). The mean Chao1 species richness for Coastal Plain, Piedmont, and Mountain regions was 4.08 ± 1.13 (±SE), 5.04 ± 1.10, and 4.83 ± 1.19, respectively. In contrast, there was a significant negative effect of latitude on species richness (*F* = 12.62; df = 1, 149; *P *< 0.01), indicating a decline in species richness along the south-to-north gradient (Table 3).

We examined the relative abundance of 4 common stink bug species (*C*. *hilaris*, *H*. *halys*, *Euschistus* complex, and *N*. *viridula*) (Table 3) to understand their spatial variation across the ecoregions. Results showed no effect of ecoregions on *C. hilaris* (*F* = 2.13; df = 2, 132; *P *= 0.12). The mean relative abundance of *C*. *hilaris* in Coastal Plain, Piedmont, and Mountain regions was 0.37 ± 0.04 (±SE), 0.29 ± 0.03, and 0.34 ± 0.05 respectively, and was not influenced by latitude (*F *= 1.76; df = 1, 132; *P *= 0.19) or elevation (*F* = 2.55; df = 1, 132; *P *= 0.11), suggesting *C*. *hilaris* broader distribution across the ecoregions (Table 3). In contrast, *H*. *halys* relative abundance varied significantly among ecoregions (*F* = 11.48; df = 2, 117; *P *< 0.01). The mean relative abundance of *H*. *halys* was lower in the Coastal Plain compared to the Piedmont, but did not differ significantly between the Piedmont and Mountain regions (Table 3). There was no effect of latitude (*F* = 1.76; df = 1, 117; *P *= 0.19) or elevation (*F* = 1.56; df = 1, 117; *P *= 0.21) on *H*. *halys* relative abundance across the ecoregions (Table 3).

The relative abundance of *Euschistus* complex did not vary among the ecoregions (*F* = 1.21; df = 2, 143; *P *= 0.30). The mean relative abundance for Coastal Plain, Piedmont, and Mountain regions was 0.26 ± 0.04 (±SE), 0.20 ± 0.02, and 0.17 ± 0.04, respectively (Table 3). Elevation also did not affect *Euschistus* complex abundance (*F* = 0.43; df = 1, 143; *P *= 0.51). In contrast, latitude had a significant and positive effect on the *Euschistus* complex (*F* = 22.26; df = 1, 143; *P *< 0.01), with increasing relative abundance along the south-to-north gradient (Table 3). Relative abundance of *N*. *viridula* did not differ among the ecoregions (*χ*^2^ = 0.91; df = 2; *P *= 0.64). In contrast, both latitude (*χ*^2^ = 27.91; df = 1; *P *< 0.01) and elevation (*χ*^2^ = 8.74; df = 1; *P *< 0.01) had a strong influence on *N*. *viridula* relative abundance, suggesting *N*. *viridula* abundance decreased along a south-to-north gradient and with an increase in elevation (Table 3).

### Economic Risk

**Fig. 3.**
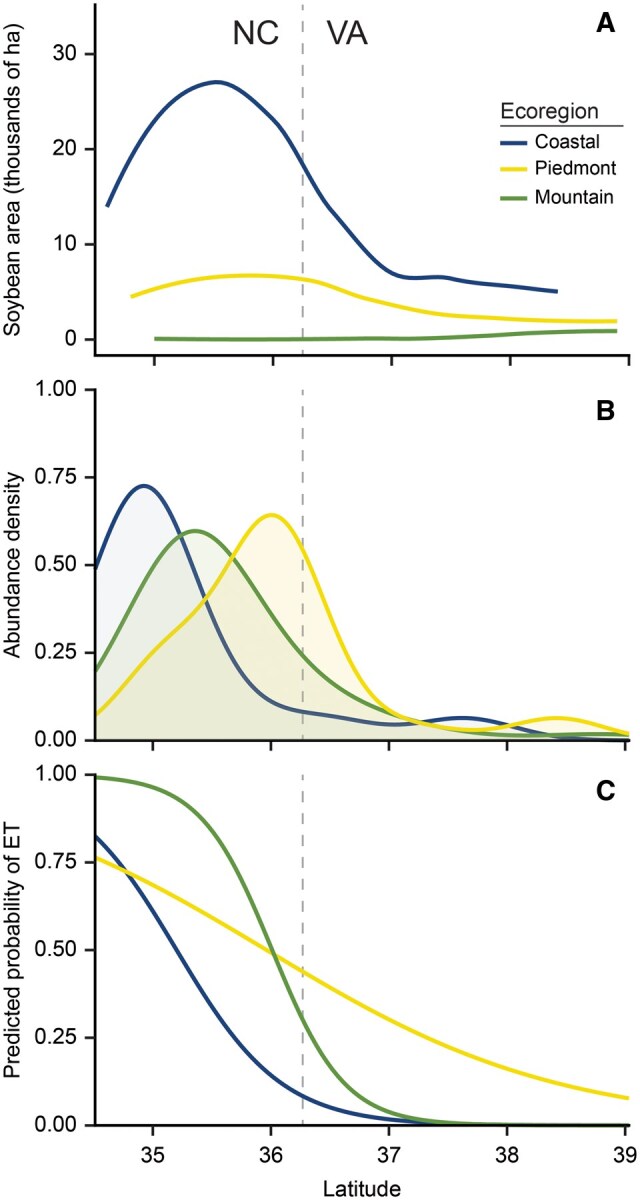
Area of soybean production by ecoregion (A). Density plot of total stink bugs abundance by ecoregions along a latitudinal gradient (B). Density values were estimated from field-level abundance and represented with relative frequency. Predicted probability of exceeding the economic threshold for stink bugs (C). Plot was smoothed over latitudes for visualization. The dotted gray line corresponds to the administrative boundary of North Carolina and Virginia.

We assessed the risk of stink bug populations exceeding the ET in soybean fields in our study region. Across the years and ecoregions, populations in 35.7% of the total fields sampled (*n* = 154) were above the recommended ET. Results indicated a significant effect of ecoregions (*χ*^2^ = 6.88; df = 2; *P* = 0.03) on the probability of exceeding ET. The predicted probability (± SE) of reaching the ET was significantly higher in the Piedmont region (0.44 ± 0.08) than in the Coastal Plain (0.09 ± 0.07) but did not differ from the Mountains (0.3070 ± 0.22). Latitude (*χ*^2^ = 9.92; df = 1; *P* < 0.01) also had a significant effect. A negative association between latitude and ET (-2.24 ± 0.71) suggested that fields at higher latitudes were less likely to exceed the threshold. Sampling year was also significant (*χ*^2^ = 5.4743; df = 2; *P* = 0.02), indicating temporal variation in the probability of exceeding the threshold. A significant interaction between ecoregion and latitudes (*χ*^2^ = 8.28; df = 2; *P* = 0.02) showed that the effect of latitude varied by ecoregion (Fig. 3). Slope contrast revealed that latitude had a stronger effect in the Mountains compared to the Piedmont (estimate = -2.45 ± 0.99; *P* = 0.04) (Fig. 3; Supplementary Table S1).

## Discussion

In this study, we documented differences in soybean stink bug communities across 3 ecoregions of North Carolina and Virginia. Our results support the hypothesis that stink bug community composition has changed since earlier soybean surveys (Deitz et al. 1976, Kamminga 2008) and varies among the ecoregions. We documented unequal abundance of soybean production among ecoregions (Fig. 3A), which may relate to differences in total stink bug density across regions and latitudes (Fig. 3B). Furthermore, we showed that the risk of reaching ET differed, with fields in the Mountain region having a very high probability of reaching ET at lower latitudes (< 36°N) (Fig. 3C). This is particularly important because growers in the Mountain region are not likely to use independent crop consultants and are less likely to scout fields. As a result, this finding reinforces the need for region-specific IPM recommendations that include regions with less intensive soybean production (Fig. 3A) but higher risk for unanticipated economic losses (Fig. 3C).

Across years and ecoregions, a total of 30 stink bug species and subspecies were identified, with *C*. *hilaris*, *H*. *halys*, *N*. *viridula*, and *E*. *servus* being the most common (Table 1). These 4 taxa were common in previous surveys from southeastern soybean (Bundy and McPherson 2000, Smith et al. 2009, Temple et al. 2013b, Bakken et al. 2015, Pilkay et al. 2015). Notably, *H*. *halys* and *N*. *viridula* were the second and third most abundant species observed in our study, respectively. Neither was reported in an earlier survey conducted in North Carolina (Deitz et al. 1976) and Virginia (Kamminga 2008). *Podisus maculiventris* was the most abundant predatory species, consistent with reports from other soybean-producing regions (McPherson and McPherson 2000, Koch and Pahs 2014, Pezzini et al. 2019). Several other stink bug species were rare (Table 1), similar to observations from other soybean-producing states (Bundy and McPherson 2000, Koch and Pahs 2014, Vyavhare et al. 2014).

Stink bug diversity and species richness did not differ among the ecoregions (Fig. 1). In contrast, stink bug species composition was significantly different across a latitudinal gradient, with both diversity and richness decreasing along a south to north gradient. This pattern aligns with a classic latitudinal gradient in arthropod diversity, commonly reported in natural ecosystem-focused studies (Hillebrand 2004, Kinlock et al. 2017). The latitudinal variation in species diversity is shaped by combinations of abiotic factors like climatic conditions, particularly temperature and rainfall (McCain and Grytnes 2010, Ernst and Buddle 2015, Chen et al. 2024), habitat structure, availability of hosts, and biotic interactions (Tscharntke et al. 2002 and references therein, Novotny et al. 2006, McCain and Grytnes 2010, Kinlock et al. 2017). For example, the occurrence of *N*. *viridula* in northern latitudes is limited by winter temperatures in other systems (Yukawa et al. 2009). However, Ferreira Santos de Aquino et al. (2019) did not observe a latitudinal gradient in stink bug diversity or their parasitoid diversity in Brazilian soybean production systems, highlighting the importance of regional surveys to document community differences.

Our analysis revealed that the most common stink bug species were unequally distributed across ecoregions (Tables 1 and 2; Fig. 2). Two endemic species, *C*. *hilaris* and the *Euschistus* complex, were broadly distributed across all ecoregions, suggesting ecological tolerance and adaptability to diverse environmental conditions (Greene et al. 2006, Temple et al. 2013b). In contrast, the invasive *H*. *halys* was generally confined to Mountain and Piedmont regions, but with lower densities in the Coastal Plain. The distribution trend was consistent with previous studies documenting *H*. *halys* abundance on non-managed host plants and soybeans in North Carolina and Virginia (Bakken et al. 2015, Ogburn et al. 2022). We observed *H*. *halys* as far east as 75°W in Virginia and 78°W in North Carolina, which provides supporting evidence of its expansion into warmer geographic regions. This variability in regional distribution may be linked to its earlier point of establishment, invasion route, and regional climatic suitability (eg Wallner et al. 2014, Cira et al. 2016, Illan et al. 2022), as well as availability of non-crop host plants—eg, *Athelia altissima* (Mill.) Swingle; *Cladrastis kentukea* (Dum.-Cours.) Rudd; *Paulownia tomentosa* (Thunb.) Sieb. & Zucc. ex Steud.—and anthropogenic structures that offer relatively stable habitats for overwintering (Lee et al. 2014, Bakken et al. 2015). However, geographic range was predicted to contract in the southeastern United States under future climate change scenarios (Zhu et al. 2024).

We also documented *N*. *viridula*, across all 3 ecoregions, with a higher relative abundance observed in the southern soybean fields (Table 3). Furthermore, we observed a decreased *N*. *viridula* abundance with elevation. This may be related to higher overwinter mortality in higher latitudes and elevations due to cold winter temperatures (Jones and Sullivan 1981, Musolin 2007, Esquivel et al. 2018). Historically, *N. viridula* was more common in southern states such as Georgia and South Carolina (Jones and Sullivan 1981, Bundy and McPherson 2000) but was absent from North Carolina (Deitz et al. 1976) and Virginia (Kamminga 2008). *Nezara viridula* is now common, with adults and nymphs sampled as far north as 37^o^N (north of the VA border), suggesting a significant northward range expansion. A similar *N. viridula* range expansion was reported in Japan, where warmer winter temperatures have facilitated the movement to higher latitudes and elevations (Musolin 2007, Tougou et al. 2009, Yukawa et al. 2009, Kiritani 2011).

The probability of soybean fields exceeding the ET varied significantly among ecoregions (Fig. 3C). Overall, about 36% of total soybean fields (55 of 154 sampled fields) exceeded ET. Over 90% of those fields were located south of 36.5°N latitude, which corresponds to the administrative boundary of North Carolina and Virginia. In addition, a large proportion of soybean acreage is concentrated below 36°N latitude in the Coastal Plain region (Fig. 3A). Soybean fields in the Piedmont region were at higher risk of exceeding ET across the study extent (Fig. 3C), while fields in the Coastal ecoregion had a lower predicted probability despite much greater soybean production area. Across the ecoregions, the probability of exceeding ET was greater at lower latitudes. Notably, the significant interaction between ecoregions and latitudes showed that the likelihood of exceeding ET along the latitudinal gradient varied among the ecoregions. The likelihood of exceeding ET was high at lower latitudes in Coastal Plain and Mountain ecoregions but decreased sharply with increasing latitudes (Fig. 3C). In contrast, this decline was more gradual in the Piedmont regions, and the shallow slope suggests that at higher latitudes the probability of exceeding ET is greater than in the other 2 regions (Fig. 3C). The stink bug density further supported the patterns in the predicted probability of exceeding the ET (Fig. 3B). Stink bug abundance density peaked at lower latitudes in Coastal Plain and Mountain regions (∼35°N), while in the Piedmont regions it peaked at higher latitudes (∼36°N) (Fig. 3B).

In the future, climate change may affect hemipteran pest phenology, voltinism, and geographic distributions (Musolin 2007, Tougou et al. 2009, Bebber et al. 2013, Chen et al. 2023, Ogburn et al. 2022, Chen et al. 2024) as well as species composition (Yukawa et al. 2007, Tougou et al. 2009, Kiritani 2011). Simultaneously, modifications to production systems, such as planting dates, tillage, and adoption of transgenic crops, may contribute to changes in stink bug composition and abundance (Panizzi and Corrêa-Ferreira 1997, Baur et al. 2000, Panizzi and Lucini 2016, Adams et al. 2017, Babu et al. 2019). Notably, North Carolina and Virginia soybean growers are adopting early soybean production systems for yield and other agronomic benefits (Morris et al. 2021, Vann et al. 2021). These shifts may support the overwintering and first-generation populations of stink bug species such as *N*. *viridula* and *Euschistus* spp., particularly in warmer regions. Earlier population development could have cascading effects on later-season crops such as cotton and later planted soybean (Jones and Sullivan 1981). Similarly, *E*. *servus* populations have been reported to increase in early-season crop hosts such as wheat and early-planted corn (Herbert and Toews 2011) before dispersing to late-planted corn and soybeans, where higher population densities were often observed (Tillman 2010). These larger infestations can create significant management challenges for growers. Stink bug species vary in their susceptibility to common insecticides (López et al. 2013, Temple et al. 2013a). Recent work documented that *E*. *servus* is twice as tolerant to bifenthrin as *N*. *viridula* and *C. hilaris* from eastern North Carolina (Panta et al. 2025, *in press*). The interspecific variability in susceptibility further reinforces the need to accurately identify and document the species to tailor field- or region-specific management recommendations.

In conclusion, stink bug community composition in North Carolina and Virginia has changed since the 1976 survey. Two species, *H*. *halys* and *N*. *viridula* are now important members of stink bug complex. We observed regional differences in probability of exceeding ET, highlighting the need for region-specific scouting and management recommendations. Our study provides baseline stink bug community composition and risk of economic damages in the North Carolina and Virginia soybeans.

## Supplementary Material

nvaf124_Supplementary_Data
