# Supplementary material for: Stink bug species composition and risk of economic damage in the southeastern soybean cropping systems
Source: Environ Entomol. 2025 Dec 4;55(1):nvaf124. doi: 10.1093/ee/nvaf124 (PMC12817314; doi:10.1093/ee/nvaf124)
Supplement: nvaf124_Supplementary_Data [file nvaf124_supplementary_data.docx]

**Title**: Stink bug species composition and risk of economic damage in the southeastern soybean cropping systems

**Authors:** Sujan Panta^1*^, George G. Kennedy^2^, Dominic D. Reisig^3^, Rachel A. Vann^4^, Benjamin L. Aigner^5^, Kyle Matthew Bekelja^6^, Sean Malone^7^, Hélène B. Doughty^8^, Tim B. Bryant^7^, Thomas P. Kuhar^5^ and Anders S. Huseth^1,9*^

Supplementary Information

Supplementary Table S1 Pairwise contrast for slope of ecoregion and latitude interaction

| Contrast | Estimate | Standard error | Z-ratio | P-value |
| --- | --- | --- | --- | --- |
| Coastal Plain-Mountain | 1.01 | 1.18 | 0.86 | 0.67 |
| Coastal Plain-Piedmont | -1.43 | 0.80 | -1.80 | 0.17 |
| Mountain-Piedmont | -2.45 | 0.99 | 2.47 | 0.04 |
